# QTL affecting fitness of hybrids between wild and cultivated soybeans in experimental fields

**DOI:** 10.1002/ece3.606

**Published:** 2013-06-05

**Authors:** Yosuke Kuroda, Akito Kaga, Norihiko Tomooka, Hiroshi Yano, Yoshitake Takada, Shin Kato, Duncan Vaughan

**Affiliations:** 1 National Institute of Agrobiological Sciences2-1-2 Kannondai, Tsukuba, Ibaraki, 305-8602, Japan; 2 Western Region Agricultural Research Center, NARO6-12-1 Nishifukatsu, Fukuyama, Hiroshima, 721-8514, Japan; 3 Tohoku Agricultural Research Center, NARO297 Uenodai Kariwano, Daisen, Akita, 019-2112, Japan

**Keywords:** Fitness, *Glycine soja* × *G. max*, introgression, QTL

## Abstract

The objective of this study was to identify quantitative trait loci (QTL) affecting fitness of hybrids between wild soybean (*Glycine soja*) and cultivated soybean (*Glycine max*). Seed dormancy and seed number, both of which are important for fitness, were evaluated by testing artificial hybrids of *G. soja* × *G. max* in a multiple-site field trial. Generally, the fitness of the F_1_ hybrids and hybrid derivatives from self-pollination was lower than that of *G. soja* due to loss of seed dormancy, whereas the fitness of hybrid derivatives with higher proportions of *G. soja* genetic background was comparable with that of *G. soja*. These differences were genetically dissected into QTL for each population. Three QTLs for seed dormancy and one QTL for total seed number were detected in the F_2_ progenies of two diverse cross combinations. At those four QTLs, the *G. max* alleles reduced seed number and severely reduced seed survival during the winter, suggesting that major genes acquired during soybean adaptation to cultivation have a selective disadvantage in natural habitats. In progenies with a higher proportion of *G. soja* genetic background, the genetic effects of the *G. max* alleles were not expressed as phenotypes because the *G. soja* alleles were dominant over the *G. max* alleles. Considering the highly inbreeding nature of these species, most hybrid derivatives would disappear quickly in early self-pollinating generations in natural habitats because of the low fitness of plants carrying *G. max* alleles.

## Introduction

Many crop species have evolved through recurrent cycles of hybridization with their wild and/or weedy relatives followed by differentiation (Harlan 1992). Gene flow from crops to their wild relatives has been commonly observed in many crop species (Ellstrand et al. 1999; Ellstrand 2003). There is a concern that transgenes in crops will persist in the gene pool of wild relatives and lead to negative environmental effects because of the difficulty in controlling gene flow completely when genetically modified (GM) crops are field planted. Possible concerns related to transgene introgression are the evolution of aggressive weeds from hybrid derivatives (Warwick et al. 2009), the influence on nontarget insects (O'Callaghan et al. 2005), and the changes in genetic diversity of wild populations (Levin et al. 1996; Lu 2008).

The probability of transgene introgression from a crop species into a wild species is largely dependent on the fitness of the F_1_ hybrid and subsequent generations. Fitness may be defined as the relative ability of an individual to survive and successfully reproduce in a given environment, with the most fit individuals leaving the greatest number of offspring (Jenczewski et al. 2003). Fitness is not only a characteristic of entire genome, it is also a property of individual genes and chromosomal segments (Harrison 1990). The persistence of transgenes from crop plants within the genomes of crop wild relatives is dependent on the fitness conferred by the transgene and by linked genomic regions (Gressel 1999; Jenczewski et al. 2003; Stewart et al. 2003). The fitness of plants carrying domestication-related genes is assumed to be lower than that of their wild relatives when tested in natural habitats (De Wet and Harlan 1975). Transgenes would be expected to disappear in natural populations when linked with domestication-related genes that lead to a selective disadvantage in wild habitats, such as seed dormancy and seed shattering (Gressel 1999; Stewart et al. 2003).

On the other hand, chromosomal blocks can introgress at a higher rate than expected when they contain advantageous gene combinations with positive fitness consequences (Rieseberg et al. 1996). There are some cases in which hybrids between wild relatives and crop plants may be as fit as or even more fit than their parents; examples have been found in *Brassica* (Snow et al. 1999; Di et al. 2009), *Raphanus* (Hovick et al. 2012), and *Sorghum* (Sahoo et al. 2010). Baack et al. (2008) found that some alleles from cultivated sunflower (*Helianthus annuus* L.) are favored in a noncrop environment and in wild genetic backgrounds. Depending on the effect of the inserted gene itself, transfer of transgenes could lead to a change in allele frequencies through a selective advantage conferred to the recipient (Hails 2000; Gepts and Papa 2003; Jenczewski et al. 2003; Snow et al. 2010; Hartman et al. 2012).

Genetically modified soybean (*Glycine max*) is economically important and accounted for 81% of the worldwide planting area of soybean in 2012 (81 million ha; James 2012). The annual wild species *Glycine soja* is found in eastern and northeastern China, Japan, Korea, and far eastern Russia (Carter et al. 2004). In Japan, *G. soja* is distributed widely in disturbed habitats such as riverbanks, roadsides, and even at the edges of soybean fields (Kaga et al. 2005; Kuroda et al. 2005, 2006b, 2007). Reproductive barriers have not been observed between *G. max* and *G. soja*, and the crosses can produce fertile F_1_ hybrids (Singh and Hymowitz 1989; Carter et al. 2004). The risk of transgene dispersal within *Glycine* is assumed to be very low in Japan because (1) outcrossing rates between *G. max* and *G. soja* are generally less than 1% (Nakayama and Yamaguchi 2002; Kuroda et al. 2008; Mizuguti et al. 2009), (2) natural F_1_ hybrids between *G. max* and *G. soja* are rare in Japan, and (3) plants derived from those hybrids survived only one to a few years in natural habitats (Kaga et al. 2005; Kuroda et al. 2005, 2006b, 2007). However, the genetic and ecological mechanisms for this lack of persistence remain unclear.

During the domestication of soybean, *G. max* evolved from *G. soja* to have large and nondormant seeds with a determinate nontwining growing habit that may affect fitness in natural habitats. The seed dormancy of wild soybean is caused by the physical structure of the seed coat, which usually does not imbibe water immediately after immersion (Rolston 1978; Ohara and Shimamoto 1994). In contrast, *G. max* bears seeds with little to no dormancy because uniform and rapid germination are important for soybean cultivation and food processing. Oka (1983) analyzed reproductive success in seminatural conditions by using hybrid derivatives between *G. max* and *G. soja* and found that plants with high seed dormancy and high seed production successfully survived. Thus, knowledge of genomic regions affecting fitness-related traits helps us to understand the reasons why hybrid derivatives between *G. max* and *G. soja* are rare in natural habitat. Although domestication-related quantitative trait loci (QTL) such as seed size and growth habit have previously been reported (e.g., Liu et al. 2007), no attempt has been made to identify genetic factors affecting the number of seeds per plant and winter seed survival in the soil.

In this study, artificial F_1_ hybrids, F_2_ populations, and backcross populations were made of two combinations of *G. soja* (W – Wild) and non-GM *G. max* (D – Domesticated); these combinations had different growth habits and represented northern and southern Japanese germplasm based on the assumption that gene flow from GM *G. max* to *G. soja* occurs in both northern and southern Japan. The degree of fitness of the hybrids and their derivatives was compared with their *G. soja* and non-GM *G. max* parents in three regions of Japan: north, central, and south. On the basis of the results, we discuss the likelihood of persistence of transgenes from *G. max* in *G. soja* populations. This is the first report of the detection of QTLs affecting fitness-related traits such as winter seed survival and seed number per plant of *G. soja* × *G. max* hybrids in experimental fields.

## Materials and Methods

### Plant materials

#### F_1_ hybrids between wild and cultivated soybean

F_1_ hybrids between *G. soja* and non-GM *G. max* were produced for two cross combinations. One combination (W1 × D1) was developed from a cross between the *G. soja* accession “JP036034” (W1) collected in Aomori Prefecture, northern Japan, and non-GM *G. max* cultivar “Ryuhou” (D1), which is widely grown in the northern Japan. The other combination (W2 × D2) was developed from a cross between *G. soja* accession “JP110755” (W2) collected in Hiroshima prefecture in southern Japan, and non-GM *G. max* “Fukuyutaka”(D2), which is widely grown in southern Japan (Table 1). The wild soybean accessions used in these crosses were obtained from the Genebank of the National Institute of Agrobiological Sciences. Non-GM *G. max* cultivars, D1 and D2, were obtained from the Tohoku Agricultural Research Center and Kyushu Okinawa Agricultural Research Center, respectively.

**Table 1:** Artificial hybrids and populations between *Glycine soja* and *G. max* evaluated in this study

Combination (*G.soja* × *G.max*)	Generation	Year	Field	Pedigree	Genetic linkage map (no. loci)	Total map length (cM)
W1^1^ × D1^2^	F_1_	2005	North^5^, Central^6^, South^7^	*G. soja* (W) × *G. max* (C)	–	–
	F_2_	2005	North, south	W × C	212	2720
	BC_1_F_1_	2006	Central	W / (W × C)	214	2609
	BC_2_F_1_	2007	Central	W / W / (W × C)	103	994
	BC_1_F_2_	2007	Central	W / (W × C)	105	931
W2^3^ × D2^4^	F_1_	2005	North, central, south	W × C	–	–
	F_2_	2005	North, south	W × C	208	2547
	BC_1_F_1_	2006	Central	W / (W × C)	199	2514
	BC_2_F_1_	2007	Central	W / W / (W × C)	72	572
	BC_1_F_2_	2007	Central	W / (W × C)	72	599

1 W1 (JP036034): *G. soja* collected from Aomori Prefecture in northern Japan.

2 D1 (Ryuhou): *G. max* cultivar commonly planted in northern Japan.

3 W2 (JP110755): *G. soja* collected from Hiroshima Prefecture in southern Japan.

4 D2 (Fukuyutaka): *G. max* cultivar commonly planted in southern Japan.

5 North field: Akita prefecture, northern Japan.

6 Central field: Ibaraki prefecture, central Japan.

7 South field: Hiroshima prefecture, southern Japan.

#### F_2_ populations

F_2_ populations, which might be expected to grow in natural habitats, were developed for testing because of the highly inbreeding nature of soybean. Two F_2_ populations, one representing the northern region (W1 × D1: 204 individuals) and the second representing the southern region (W2 × D2: 204 individuals) were developed from seeds by self-pollination of a single F_1_ hybrid plant per cross (Table 1).

#### Backcross populations

**Figure 1:**
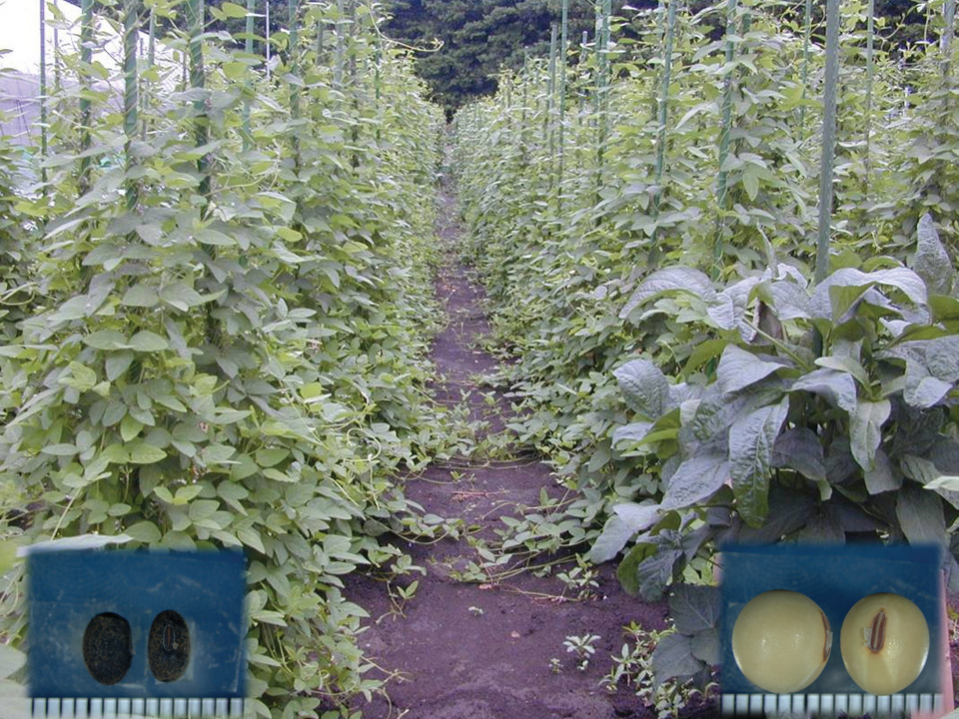
Morphological differences in wild soybean (*Glycine soja*, W2) with cultivated soybean (*G. max*, D2); Twining growing habit and small blackish seeds. Photo: Akito Kaga.

To confirm the effect of *G. max* genes in a predominantly *G. soja* background, backcross (BC) populations were developed for both W1 × D1 and W2 × D2 combinations using *G. soja* as the recurrent parent (Table 1, Fig. 1). Two BC_1_F_1_ populations (W1 × D1: 68 individuals; W2 × D2: 160 individuals) were obtained from crossing each F_1_ hybrid (donor plant) to the corresponding *G. soja* accession (recurrent parent). The success of the crossing was confirmed using 60 simple sequence repeat (SSR) markers composed of three markers per linkage group for all 20 linkage groups. Furthermore, two BC_2_F_1_ populations (W1 × D1: 60 individuals; W2 × D2: 40 individuals) were developed by crossing selected BC_1_F_1_ plants (one plant per population) to *G. soja*. The selection of the BC_1_F_1_ plants, having major *G. max* QTLs for seed dormancy and total seed number identified in F_2_ populations, was based on the genotypes of the BC_1_F_1_ populations. To investigate the fitness of the populations after an additional generation of self-pollination, the seeds obtained from self-pollination of the selected BC_1_F_1_ plants were used to develop two BC_1_F_2_ populations (W1 × D1: 150 individuals; W2 × D2: 150 individuals).

### Field locations

Among 204 F_2_ plants in the W1 × D1 population, 104 F_2_ plants, together with the 10 parents and several F_1_ hybrid (Table 1), were grown at 1 m × 1 m spacing at the Tohoku Agricultural Research Center (39.5°N, 140.4°E, Akita Prefecture, northern Japan, Appendices A1 and A2), hereafter referred to as the “north field.” The other 100 F_2_ plants, together with the parents and F_1_ hybrid, were grown at the same density at the Western Region Agricultural Research Center (34.5°N, 133.4°E, Hiroshima prefecture, southern Japan, Appendices A1 and A2), hereafter referred to as the “south field.” The W2 × D2 population, composed of 204 F_2_ plants, was grown in the north and south fields in the same manner as the W1 × D1 population (Table 1). As maintenance and evaluation of many climbing plants with a higher *G. soja* background are difficult, backcross populations (BC_1_F_1_, BC_2_F_1_, and BC_1_F_2_) were only grown at 1 m × 1 m density at the National Institute of Agrobiological Sciences (36.0°N, 140.1°E, Ibaraki Prefecture, central Japan, Appendices A1 and A2), hereafter referred to as the ‘central field’. Seed coats were scratched with a razor blade and germinated in a small pot at the beginning of July in 2005, at the middle of June in 2006, and at the end of May for in 2007. The seedlings were transplanted to the field in the middle of July every year. Three stakes with a net strung between the stakes per plant were used to guide twining stems. During October to November, mature pods with seeds were harvested by hand twice a week. Standard agricultural practices such as applications of fertilizer (650 kg/ha of 3 parts nitrogen, 10 parts phosphate, and 10 parts potassium; 1000 kg/ha of fused magnesium phosphate; 1000 kg/ha of limestone), weeding, insecticides to control stink bug and common cutworm, were conducted.

### Trait measurement

Of a total of 11 fitness-related traits (Table 2), 10 were treated as quantitative traits and one (seed coat color) was treated as a qualitative trait. Two seed dormancy–related traits, namely seed winter survival (DORM_1) and seed hardness (DORM_2), were evaluated using the seeds from individual plants. As germination of all the hard seeds from randomly selected lines were confirmed by the mechanical abrasion on the wetted filter paper or soil at room temperature, hard seeds were treated as viable nongerminated seeds. Seed production–related traits, namely total seed number (PROD_1), seed total weight (PROD_2), 100-seed weight (PROD_3), total pod number (PROD_4), stem dry weight (PROD_5), and stem length (PROD_6) were evaluated for each plant (Table 2). Those traits were recorded on a per plant basis after the seeds of each plant had matured. As flowering of the two southern accessions (W2 and D2) as well as F_1_ plants and most of the W2 × D2 F_2_ plants were late flowering in the north field, those whole plants (7 of 10 W2, all 5 D2, all 5 of the W2 × D2 F_1_, and 73 of 104 W2 × D2 F_2_ plants) were taken from the field before the first snowfall and dried in the greenhouse to obtain mature seeds. The methods of trait evaluation in the backcrossing populations were the same as for the F_2_ populations. The number of days from sowing to first flowering was recorded as FLOW. The total number of seeds expected to germinate in the following year (SURV) was estimated as PROD_1 multiplied by DORM_1. The mean and standard deviation for each trait and the correlation coefficient between each pair of traits were calculated. Differences in mean values between *G. soja*, *G. max*, and F_1_ hybrids were analyzed separately for each field location in each year with the Mann–Whitney *U*-test or Kruskal–Wallis test. The median and range instead of mean and standard deviation are reported for segregating populations. All statistical analyses were conducted using R version 2.9.2 (R Development Core Team 2009).

**Table 2:** Fitness-related traits evaluated in this study

General attribute	Trait (unit)	Abbreviation	Evaluation method
Seed dormancy	Seed winter survival (%)	DORM_1	Percentage of germinated and viable nongerminated seeds after burial of mesh bags containing 20 unscarified seeds 3 cm below the soil surface of each experimental field (north, central, or south) from late December to the following spring (April–May)
	Seed hardness (%)	DORM_2	Percentage of nonimbibed seeds after soaking for 4 days in an incubator at 4°C
	Seed coat color	DORM_3	Black or other (buff, green, or brown)
Seed production	Total seed number	PROD_1	Total number of harvested seeds
	Total seed weight (g)	PROD_2	Total weight of harvested seeds
	100-seed weight (g)	PROD_3	100-seed weight
	Total pod number	PROD_4	Total number of harvested pods
	Stem dry weight (g)	PROD_5	Stem weight after drying for 7 days at 70°C
	Stem length (cm)	PROD_6	Length from ground to top of stem
Seed dormancy & production	Total number of seeds expected to germinate in the following year	SURV	Total number of seeds (PROD_1) multiplied by the winter seed survival rate (DORM_1)
Flowering phenology	Days to first flower (day)	FLOW	Number of days from sowing to flowering of first flower

### Genotyping

Total DNA of each putative F_1_ seed was extracted from a small piece of cotyledon tissue using an EZ1 DNA Tissue kit (Qiagen, Tokyo, Japan). Total DNA of F_2,_ BC_1_F_1,_ BC_2_F_1_, and BC_1_F_2_ individuals was extracted from 100 mg of fresh leaf tissue. DNA concentration was adjusted between 5 and 25 ng/μL by comparing with known concentrations of standard λ DNA on a 1.5% agarose gel. A total of 720 SSR markers from SoyBase (http://soybase.org/) were screened to detect polymorphisms between the parents. Five markers were also included to track the three classical soybean loci, *I*, *T*, and *Dt1* (Appendix A3). Three markers, *dCHS1* (Matsumura et al. 2005), *AY262686B*, and *AY262686Z,* were used to track the *I* locus, which controls seed coat color and might be related to seed winter survival. A single-base indel marker *sF3′H1* reported by Toda et al. (2002) was used to detect the *T* locus, a locus that controls pubescence color and interacts with the *I* locus. A SSR marker *LFsoy3* was designed to track the *Dt1* locus, which might be related to stem length and seed total number. These markers were amplified by using KOD-plus polymerase (Toyobo, Osaka, Japan), based on the manufacturer's guide, in a GeneAmp 9700 PCR system (Applied Biosystems, Tokyo, Japan). Polymorphisms were scored by using banding patterns in 12% polyacrylamide gel.

Successful crossing was confirmed by analysis of DNA from putative F_1_ seeds, based on the genotype of the polymorphic SSR marker *Satt207*, which has a different allele in each of the four parents (W1, 177 bp; D1, 234 bp; W2, 210 bp; and D2, 231 bp). To genotype F_2_, BC_1_F_1_, BC_2_F_1_, and BC_1_F_2_ individuals, polymorphic markers were selected at about 20-cM intervals based on the composite map of soybean from SoyBase (http://soybase.org/). Using four types of fluorescent labels (6-FAM, VIC, NED, or PET), multiplex PCR was performed to detect segregation patterns within each population. The PCR reaction mixture consisted of a total volume of 5 μL, containing 1.7 μL of template DNA, 2.5 μL of 2 × Qiagen Multiplex PCR Master Mix, 0.5 μL of a four-primer mix (1.25 μmol/L each), and 0.3 μL of water. PCR amplification was perform in a GeneAmp 9700 (Applied Biosystems) or iCycler (BioRad, Tokyo, Japan) thermal cycler programmed with an initial activation step at 95°C for 15 min; followed by 40 cycles of 30 sec at 94°C for denaturation, 90 sec at 57°C for annealing, and 60 sec at 72°C for extension; followed by 30 min at 60°C for final extension. For analysis, 3 μL of PCR product was denatured at 95°C for 5 min after mixing with 10 μL of Hi-Di formamide (Applied Biosystems) and 15 nL of GeneScan-500LIZ size standard (Applied Biosystems). Denatured samples were analyzed by using a 3100 Genetic Analyser (Applied Biosystems) and the output was analyzed using Gene Mapper 3.0 software (Applied Biosystems).

### Linkage map construction

Linkage maps were constructed for F_2,_ BC_1_F_1,_ BC_2_F_1_, and BC_1_F_2_ populations by using Joinmap ver. 3.0 software (Van Ooijen and Voorrips 2001) according to the method of Han et al. (2005). The recombination frequencies were converted into map distances using the Kosambi mapping function (Kosambi 1944).

### QTL analysis

The QTL analysis for phenotypic data from the BC_1_F_1,_ BC_2_F_1_, and BC_1_F_2_ individuals was conducted with MultiQTL ver. 2.6 software according to Peng et al. (2003). For phenotypic data of the F_2_ individuals from the two field environments (north and south), a single QTL with multiple environment model was fitted to scan the entire genome (Korol et al. 1998, 2001). Statistical significance thresholds (α = 0.05) for putative QTLs were tested by 10,000 runs of a permutation test (Churchill and Doerge 1994). Multiple interval mapping (Kao et al. 1999) was then conducted to reduce the background variation by taking into account QTL effects from other chromosomes. After the permutation test runs, the parameters of significant QTLs (statistical thresholds α = 0.05) were reported as position, additive and dominant effects, and percentage of variance explained (PVE).

## Results

### Fitness of hybrids and their derivatives

#### Cultivated and wild soybean

The following domestication-related traits generally differed between the *G. soja* and *G. max* parents for both combinations (W1 × D1 and W2 × D2) tested at all three field locations (north, central, and south) in 2005 (Table 3). The means of *G. max* were higher than those of *G. soja* for PROD_3, whereas the means of *G. soja* were generally higher than those of *G. max* for DORM_1, DORM_2, PROD_1, PROD_4, PROD_6, SURV, and FLOW. In contrast, PROD_2 and PROD_5 were not notably different between *G. soja* and *G. max*. Especially, the means of *G. max* for PROD_2 tended to be similar to or higher than those of *G. soja* at their recommended regions for growing. Although no *G. max* data were obtained from the south field in 2005, we confirmed these trends in 2004 (A. Kaga & Y. Kuroda, unpublished data).

**Table 3:** Summary of phenotypic values of *G. max*, *G. soja*, F_1_, F_2_, BC_1_F_1_, BC_1_F_2_, and BC_2_F_1_ populations

Combination	Field location (year)		No. individual		DORM_		PROD_							
			
					1	2	1	2	3	4	5	6	SURV	FLOW
W1 × D1	North (2005)	W1	9	Mean (SD)	97.5^a^ (3.5)	95.0^a^ (4.1)	421^ab^ (203)	14.0^b^ (7.5)	3.3^c^ (0.3)	326^ab^ (193)	9.5^ab^ (6.1)	188.2^a^ (28.2)	410^a^ (197)	55.8^a^ (2.2)
		D1	6	Mean (SD)	0.0^b^ (0.0)	0.0^b^ (0.0)	174^b^ (27)	54.6^a^ (8.3)	31.4^a^ (1.7)	75^b^ (11)	6.6^b^ (0.9)	23.0^b^ (1.1)	0^b^ (0)	37.0^c^ (0.0)
		F_1_	3	Mean (SD)	55.0^ab^ (0.0)	83.3^a^ (5.8)	688^a^ (241)	63.6^a^ (21.4)	9.3^ab^ (0.2)	676^a^ (73)	31.9^a^ (10.3)	176.0^a^ (25.2)	378^a^ (133)	53.0^ab^ (2.6)
		F_2_	104 (103)^1^	Median (range)	55.0 (0.0–100.0)	70.0 (0.0–100.0)	368 (48–883)	32.0 (3.1–81.0)	8.6 (4.4–13.9	328 (53–1426)	14.4 (1.3–48.4)	109.0 (25.0–272.0)	187 (0–876)	49.0 (37.0–63.0)
	Central (2005)	W1	7	Mean (SD)	–	67.1^a^ (13.8)	1211^a^ (297)	41.0^a^ (10.7)	3.4^b^ (0.3)	717^a^ (199)	41.9^a^ (8.4)	187.0^a^ (36.7)	–	50.3^a^ (1.0)
		D1	9	Mean (SD)	–	0.0^b^ (0.0)	50^b^ (21)	14.6^b^ (6.1)	29.2^a^ (1.4)	66^b^ (14)	25.6^b^ (2.9)	29.1^b^ (1.4)	–	34.2^b^ (0.4)
		F_1_	1	Mean (SD)	–	40.0^1^^ab^ (–)	636^1^^ab^ (–)	70.7^1^^ab^ (–)	11.1^1ab^ (–)	389^1^^ab^ (–)	45.8^1^^ab^ (–)	110.0^1^ ^ab^ (–)	–	42.0^1^^ab^ (–)
	South (2005)	W1	5	Mean (SD)	100.0^ns^ (0.0)	91.0^a^ (8.9)	1049^ns^ (561)	44.9^ns^ (26.3)	3.8^b^ (0.6)	575^ns^ (205)	35.8^ns^ (15.8)	116.0^ns^ (89.0)	1049^ns^ (561)	44.2^ns^ (1.6)
		D1	0	Mean (SD)	–	–	–	–	–	–	–	–	–	–
		F_1_	6	Mean (SD)	17.5^ns^ (3.5)	34.2^b^ (17.7)	796^ns^ (290)	77.8^ns^ (28.5)	9.8^a^ (0.4)	424^ns^ (140)	23.3^ns^ (6.7)	134.3^ns^ (26.7)	139^ns^ (51)	45.3^ns^ (3.1)
		F_2_	100 (96) 1	Median (range)	27.5 (0.0-100.0)	50.0 (0.0–100.0)	517 (2–2109)	54.2 (0.3–217.2)	9.7 (3.8–13.9)	302 (31–1369)	19.0 (2.0–69.0)	141.5 (24.0–249.0)	128 (0–1673)	43.0 (31.0–48.0)
	Central (2006)	W1	9	Mean (SD)	96.7^a^ (2.9)	100.0^a^ (0.0)	2674^a^ (808)	59.1^ns^ (17.1)	2.2^b^ (0.2)	823^a^ (244)	43.0^a^ (17.1)	303.8^a^ (55.1)	2585^a^ (781)	53.7^a^ (1.8)
		D1	10	Mean (SD)	20.0^b^ (20.0)	0.0^b^ (0.0)	111^b^ (9)	11.4^ns^ (2.3)	23.5^a^ (3.1)	29^b^ (5)	31.7^b^ (8.7)	27.7^b^ (2.3)	0^b^ (0)	40.1^b^ (0.7)
		BC_1_F_1_	66	Median (range)	95.0 (0.0–100.0)	95.0 (40.0–100.0)	2588 (617–4550)	89.8 (9.3–162.7)	3.5 (1.4–5.4)	835 (194–1454)	36.8 (7.2–70.9)	300.0 (90.0–435.0)	2166 (0–4416)	51.0 (45.0–67.0)
	Central (2007)	W1	10	Mean (SD)	100.0^ns^ (0.0)	100.0^a^ (0.0)	5627^a^ (696)	177.9^ns^ (19.2)	3.2^b^ (0.1)	1788^a^ (258)	334.1^a^ (32.0)	372.0^a^ (25.8)	5627^a^ (696)	79.6^a^ (2.9)
		D1	9	Mean (SD)	0.0^ns^ (0.0)	5.0^b^ (7.1)	81^b^ (24)	22.9^ns^ (6.7)	28.5^a^ (1.3)	40^b^ (12)	30.9^b^ (8.8)	24.3^b^ (4.1)	0^b^ (0)	72.0^b^ (1.8)
		BC_2_F_1_	150	Median (range)	100.0 (60.0–100.0)	95.0 (45.0–100.0)	5141 (2682–7297)	199.7 (112.1–277.6)	4.0 (3.3–4.8)	1634 (925–2413)	314.7 (161.5–465.6)	375.0 (260.0–460.0)	5123 (2150–7297)	82.0 (74.0–89.0)
		BC_1_F_2_	60	Median (range)	80.0 (0.0–100.0)	85.0 (0.0–100.0)	3655 (218–5878)	186.1 (14.4–294.2)	5.1 (3.4–8.8)	1216 (81–2027)	240.8 (16.7–462.9)	270.0 (45.0–415.0)	2714 (0–5746)	80.0 (72.0–92.0)
W2 × D2	North (2005)	W2	10	Mean (SD)	77.5^ns^ (10.6)	93.3^a^ (2.6)	1418^a^ (735)	32.1^b^ (17.7)	2.2^c^ (0.1)	1034^a^ (489)	31.8^ns^ (12.8)	147.0^ns^ (45.2)	1099^ns^ (570)	71.2^a^ (1.1)
		D2	6 (0) 1	Mean (SD)	–	0.0^b^ (0.0)	122^b^ (56)	21.2^b^ (11.0)	17.1^a^ (1.6)	356^b^ (36)	51.8^ns^ (5.9)	69.2^ns^ (6.7)	–	56.0^c^ (0.0)
		F_1_	5	Mean (SD)	25.0^ns^ (7.1)	15.0^ab^ (6.1)	1076^a^ (320)	86.6^a^ (27.4)	8.0^ab^ (0.4)	796^a^ (295)	45.8^ns^ (18.8)	129.0^ns^ (74.1)	269^ns^ (80)	65.0^b^ (2.7)
		F_2_	104 (102) 1	Median (range)	17.5 (0.0–100.0)	5.0 (0.0–90.0)	723(88–1694)	53.7 (2.9–125.0)	7.5 (2.5–11.4)	710 (250–2040)	41.4 (14.9–106.0)	111.0 (30.0–298.0)	144 (0–989)	63.0 (58.0–85.0)
	Central (2005)	W2	6	Mean (SD)	–	94.0^a^ (5.5)	1319^a^ (217)	42.1^b^ (7.6)	3.2^b^ (0.2)	1282^a^ (404)	50.4^ns^ (6.9)	117.3^a^ (16.3)	–	66.7^a^ (0.5)
		D2	7	Mean (SD)	–	0.0^c^ (0.0)	100^b^ (31)	23.7^c^ (7.6)	23.7^a^ (2.1)	216^b^ (58)	58.5^ns^ (9.4)	49.9^b^ (5.5)	–	53.4^b^ (1.1)
		F_1_	4	Mean (SD)	–	42.5^ab^ (15.0)	1012^ab^ (156)	99.2^a^ (17.2)	9.8^ab^ (0.4)	942^ab^ (419)	64.5^ns^ (18.5)	119.5^a^ (13.8)	–	58.8^ab^ (1.0)
	South (2005)	W2	2	Mean (SD)	100.0^ns^ (0.0)	87.5^ns^ (10.6)	5137^1^^ns^ (–)	143.3^1^^ns^ (–)	2.8^1^^ns^ (–)	1714^1^^ns^ (–)	54.0^1^^ns^ (–)	104.0^1^^ns^ (–)	5137^ns^(–)	65.0^1^^ns^ (–)
		D2	2	Mean (SD)	–	–	–	–	–	–	–	–	–	–
		F_1_	5	Mean (SD)	10.0^ns^ (0.0)	9.0^ns^ (4.2)	2744^ns^ (234)	258.8^ns^ (23.4)	9.4^ns^ (0.4)	1354^ns^ (138)	68.4^ns^ (11.1)	150.2^ns^ (41.9)	274^ns^ (23)	54.8^ns^ (1.6)
		F_2_	100	Median (range)	0.0 (0.0–100.0)	7.5 (0.0–100.0)	1874 (138–4694)	186.9 (13.4–468.5)	9.7 (5.0–18.1)	1051 (194–2450)	53.5 (13.0–135.0)	142.5 (30.0–282.0)	0 (0–3620)	53.0 (49.0–62.0)
	Central (2006)	W2	10	Mean (SD)	98.3^a^ (2.6)	92.5^a^ (3.5)	4189^a^ (804)	104.6^ns^ (24.3)	2.5^b^ (0.3)	1354^a^ (274)	62.6^ns^ (15.4)	251.9^ns^ (30.1)	4119^a^ (791)	83.7^a^ (1.3)
		D2	10	Mean (SD)	10.0^b^ (12.6)	0.0^b^ (0.0)	326^b^ (48)	106.1^ns^ (18.6)	32.4^a^ (1.5)	157^b^ (25)	30.7^ns^ (4.3)	44.5^ns^ (2.0)	33^b^ (5)	63.5^b^ (1.2)
		BC_1_F_1_	160	Median (range)	100.0 (0.0–100.0)	100.0 (20.0–100.0)	3973 (328–7574)	164.1 (14.6–320.0)	4.1 (2.4–5.3)	1289 (109–2443)	75.1 (31.8–145.5)	280.0 (190.0–380.0)	3801 (0–7574)	79.0 (73.0–86.0)
	Central (2007)	W2	10	Mean (SD)	100.0^a^ (0.0)	92.5^ns^ (3.5)	6016^a^ (1764)	178.6^ns^ (50.0)	3.0^b^ (0.2)	2073^a^ (615)	327.4^a^ (57.4)	335.5^a^ (33.0)	6016^a^ (1764)	105.1^a^ (1.7)
		D2	10	Mean (SD)	0.0^b^ (0.0)	0.0^ns^ (0.0)	439^b^ (54.0)	120.7^ns^ (17.8)	27.7^a^ (2.1)	220^b^ (27)	123.2^b^ (12.3)	45.9^b^ (6.4)	0^b^ (0.0)	88.5^b^ (1.1)
		BC_2_F_1_	149	Median (range)	100.0 (30.0–100.0)	95.0 (5.0–100.0)	5863 (4298–9784)	196.7 (126.0–311.6)	3.2 (2.4–3.9)	1999 (1496–3494)	326.1 (211.4–498.8)	300.0 (250.0–420.0)	5673 (1444–9784)	106.0 (103.0–109.0)
		BC_1_F_2_	40	Median (range)	100.0 (20.0–100.0)	95.0 (15.0–100.0)	4244 (1511–7806)	166.9 (48.3–292.9)	3.9 (2.9–5.2)	1429 (521–2692)	255.1 (75.2–472.8)	275.0 (80.0–375.0)	4002 (934–7806)	105.0 (100.0–109.0)

Different alphabet among parents and populations at each field location indicates significant difference at 5% level by Mann–Whitney's U-test or Kruskal–Wallis test.

1 Trait abbreviations are as defined in Table 2. –, no data recorded.

Number of seed-producing individuals.

#### F_1_ hybrids and F_2_ populations

**Figure 2:**
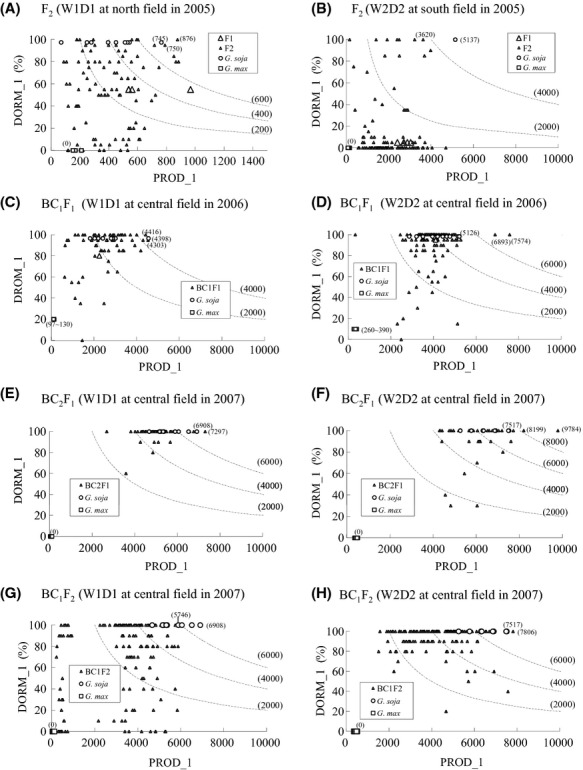
Distribution of SURV, calculated by multiplying DORM_1 and PROD_1 for each combination (W1D1 or W2D2) and generation (F_2_, BC_1_F_1_, BC_2_F_1_, or BC_1_F_2_). Numbers in brackets indicate SURV values; areas between dotted lines indicate ranges of SURV values.

The phenotypic values of the F_1_ and F_2_ generations in most field locations were intermediate between *G. soja* and *G. max* for DORM_1, DORM_2, PROD_1, PROD_3, PROD_4, PROD_6, SURV, and FLOW (Table 3, Fig. 2A and B). However, the means of PROD_2 and PROD_5 in the F_1_ and F_2_ generations tended to be similar to or higher than those of *G. soja* at the recommended regions for growing the *G. max* parent. Most of the *G. soja* seeds dug up from the soil in the spring did not imbibe water, whereas the *G. max* seeds were rotten. Seeds from F_1_ and F_2_ plants were of all types: hard seeds that did not absorb water, water-absorbing viable seeds, and rotten seeds. DORM_2 was positively correlated with DORM_1 (*P* < 0.05) in the F_2_ generations of W1 × D1 (seeds harvested from the north field, *R*^2^ = 0.81; seeds harvested from the south field, *R*^2^ = 0.69, Appendix A4) and W2 × D2 (seeds harvested from the north field, *R*^2^ = 0.85; seeds harvested from the south field, *R*^2^ = 0.60).

The extent of DORM_1 was associated with maternal-inherited seed coat color and the pubescence color of the F_3_ seeds produced on F_2_ plants (Table 4). *G. soja* has black seeds and brown pubescence, and *G. max* has yellow seeds and white pubescence. High DORM_1 was observed for seeds with black or brown seed coat color produced by F_2_ plants with brown pubescence color, and most of those seeds did not imbibe water when tested in the spring (brown seeds, 75.9%; black seeds, 75.5%). The seeds with other colors of pubescence had relatively low DORM_1. In particular, the seeds with brown seed coat color produced by F_2_ plants with white pubescence color (22 of 27 F_2_ plants) were severely cracked or split and could not be found in the following spring (DORM_1, 0.2%).

**Table 4:** Relationships between percentage of seed winter survival (DORM_1) and colors of seed coat and pubescence in F_2_ populations

Combination	Seed production location		Seed coat color (pubescence color)					
	
			Black (brown)	Brown (white)	Brown (brown)	Yellow (unclassified)	Green (unclassified)	Unclassified
			[*i*/*i*, *T*/−, *R*/−]^1^	[*i*/*i*, *t*/*t*, *r*/*r*]	[*i*/*i*, *T*/−, *R*/−]	[*I*/−2,,]	[*I*/−2,,]	
W1 × D1	North	DORM_1 (%)	92.7 ± 11.8^a^	0.0 ± 0.0^b^	81.0 ± 12.4^ab^	33.1 ± 25.5^b^	51.8 ± 29.2^ab^	43.0 ± 35.6^b^
		*n*	15	4 (4)^3^	6	19 (1)^3^	54	5
	South	DORM_1 (%)	83.1 ± 17.4^a^	0.0 ± 0.0^b^	95.8 ± 4.9^a^	12.9 ± 14.2^b^	32.0 ± 28.4^b^	–
		*n*	17	8 (4)^3^	6	13 (1)^3^	52 (5)^3^	0
W2 × D2	North	DORM_1 (%)	51.3 ± 28.7^a^	0.0 ± 0.0^c^	57.5 ± 32.3^ab^	14.0 ± 19.2^abc^	15.9 ± 19.3^bc^	37.5 ± 23.2^abc^
		*n*	16	6 (6)^3^	4	11 (3)^3^	55 (1)^3^	10
	South	DORM_1 (%)	74.2 ± 28.4^a^	0.6 ± 1.7^b^	64.2 ± 44.4^a^	4.2 ± 8.4^b^	4.2 ± 10.6^b^	–
		*n*	13	9 (6)^3^	6	24	48 (4)^3^	0
Overall		DORM_1 (%)	75.5 ± 26.9^a^	0.2 ± 1.0^c^	75.9 ± 30.4^a^	15.6 ± 20.8^bc^	26.5 ± 29.6^b^	42.1 ± 27.7^ab^
		*n*	61	27 (22)^3^	22	68 (3)^3^	209 (10)^3^	15

Different alphabet among genotypes at each field location indicates significant difference at 5% level by Kruskal–Wallis test.

1 Presumed genotypes at the *I*, *T*, and *R* loci.

2 Genotype at the *I* locus would be *I*/*i*^*i*^ in the W2 x D2 population.

3 Number of cracked or split seeds.

The PROD_1 of the F_1_ plants was generally intermediate between *G. soja* and *G. max* for both the W1 × D1 and W2 × D2 combinations (Table 3, Fig. 2A and B). An exception was found in the north field, where PROD_1 of the F_1_ plants from the W1 × D1 combination (average 688) was similar to or higher than that of the *G. soja* parent (average 421). The mean values of PROD_2 and PROD_5 in the F_1_ generation were also higher than those of the parents. In the next generation, PROD_1 of several F_2_ individuals was similar to or higher than that of the *G. soja* parent. This transgressive growth of PROD_1 may be explained by heterosis or positional effect within a field for plant size–related traits because of significant (*P* < 0.05) positive correlations between PROD_1 and plant size–related traits such as PROD_5 and PROD_6 (Appendix A4).

The values for SURV of *G. soja* and *G. max* were different within both the W1 × D1 combination and the W2 × D2 combination because *G. soja* had both high PROD_1 and DORM_1, whereas *G. max* had low PROD_1 and zero DORM_1 (Table 3, Fig. 2A and B). Average SURV of F_1_ plants was intermediate between *G. soja* and *G. max* for each combination. Greater variation was observed in the F_2_ progenies than in the F_1_ plants because of genetic segregation of PROD_1 and DORM_1.

#### Backcross populations

For the backcross populations (BCs; BC_1_F_1_, BC_2_F_1_, and BC_1_F_2_) from both combinations, plants were grown only in the central field in 2006 and 2007. The phenotypic differentiation between *G. soja* and *G. max* in the central field was similar to that seen in the other fields in 2005. All trait values of the BCs were clearly shifted toward those of the *G. soja* recurrent parents. For both combinations, the medians of the BC_1_F_1_ and BC_2_F_1_ populations were very close to the means of *G. soja* for all traits (Table 3). In contrast, the extent of shift in BC_1_F_2_ populations for DORM_1, DORM_2, PROD_1, and SURV_1 was not obvious as in the BC_1_F_1_ and BC_2_F_1_ populations. The plant type of the backcross generations was vigorous in both 2006 and 2007, when mulch sheets were used on the surface of soil; in contrast, the F_1_ and F_2_ generations, which were grown without the sheets in 2005, were less vigorous.

Because *G. soja* had higher PROD_1 and DORM_1 than *G. max*, SURV of *G. soja* was higher than that of *G. max* in both the W1 × D1 and W2 × D2 combinations in all three backcross generations (Table 3, Fig. 2C–H). The medians of SURV in the BC_1_F_1_ and BC_2_F_1_ generations were very close to *G. soja*; still, there was variation in both DORM_1 and PROD_1 in the BC_1_F_1_ and BC_2_F_1_ generations (Fig. 2C–H). Some individuals had the potential to yield large numbers of dormant seed because the number of seeds (PROD_1) was greater than *G. soja* and the seed dormancy (DORM_1) was similar.

### QTL analysis for F_2_ populations

**Figure 3:**
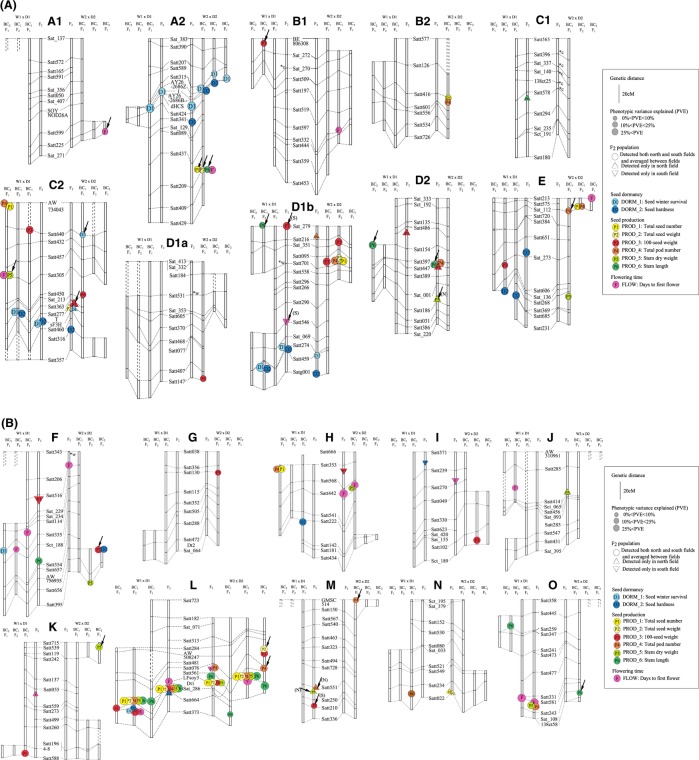
(A) Summary of fitness-related QTLs detected in F_2_, BC_1_F_1_, BC_2_F_1_, and BC_1_F_2_ generations of both north (W1 × D1) and south (W2 × D2) combinations of *Glycine soja* × *G. max* hybrids for linkage group (A1–E). *w and *c indicate *G. soja* (wild) and *G. max* (cultivated) homozygote excess, respectively, at the designated SSR locus. Arrows indicate QTLs with allelic effects opposite of those predicted by the parental phenotype for traits differing between *G. soja* and *G. max*. (N) or (S) next to an arrow indicates that the effect was observed only in the north field or south field, respectively.

Of 720 markers screened, 359 and 378 markers revealed clear polymorphisms between *G. soja* and *G. max* in the W1 × D1 and W2 × D2 populations, respectively. Of these, 212 and 208 markers were used to develop F_2_ linkage maps of the W1 × D1 and W2 × D2 populations, respectively (Table 1, Fig. 3). Although gaps of more than 30 cM were observed between *Satt285* and *Satt414* on LG-J over populations and generations, the SSR markers were otherwise distributed evenly across the soybean genome, and marker orders were conserved between the W1 × D1 and W2 × D2 population maps as well as between those maps and the composite map by Song et al. (2004). The total lengths of the linkage maps developed here were about 2500 cM for the F_2_ and BC_1_F_1_ populations, comparable to the lengths of the SSR-based linkage maps developed by Song et al. (2004) (2524 cM) and Liu et al. (2007) (2383 cM).

Several markers (1.4% and 3.4% of the markers in the W1 × D1 and W2 × D2 populations, respectively) showed segregation ratios significantly (*P* < 0.05) deviated from the expected 1:2:1 ratio of *G. soja* homozygote, heterozygote, and *G. max* homozygote. Although most markers with segregation distortion were scattered over several linkage groups and were not consistent between the W1 × D1 and W2 × D2 populations, five of the distorted markers were adjacent and located in the upper half of LG-C1 in the W2 × D2 population (Fig. 3A). Paracentric inversions and reciprocal translocations, which can lead to pollen and ovule sterilities and have been found between a specific Chinese accession of *G. soja* and *G. max* (Singh and Hymowitz 1988; Palmer et al. 2000), might account for the segregation distortions in these Japanese germplasm sources as well.

In total, 28 and 27 QTLs related to seed dormancy, seed production, and flowering phenology were detected in the F_2_ generation of W1 × D1 and W2 × D2 populations, respectively (Fig. 3, Appendix A5). Among them, QTLs in three regions (LG-A2, -C2, and -D1b) had large effects on seed dormancy and QTL in one region (LG-L) had a significant effect on seed production.

#### Seed dormancy

Eight and 6 QTLs associated with seed dormancy were detected in the W1 × D1 and W2 × D2 populations, respectively (Fig. 3, Appendix A5). The *G. max* alleles at all of those QTLs had additive effects (Add.) of decreasing DORM_1 (Add, –3 to –37%; PVE, 6.4–76.2%) and DORM_2 (Add, –1 to –26%; PVE, 6.2–42.7%). Three major QTLs, which were located on LG-A2, -C2, and -D1b, were associated with DORM_1 and DORM_2 in both populations (Fig. 3A). The *G. max* allele at the QTL on LG-A2 had larger additive effects in the W2 × D2 population than in the W1 × D1 population. The QTL on LG-A2 was located near the *I* locus, and the QTL on LG-C2 was close to the *T* locus. The additive effect of the QTL on LG-A2 detected in seeds harvested from the south field tended to be higher than that for seeds harvested from the north field. In contrast, the additive effect of the QTL on LG-C2 and LG-D1b detected in seeds harvested from the south field tended to be lower than that for seeds harvested from the north field.

#### Seed production

Sixteen and 17 QTLs related to seed production were detected in W1 × D1 and W2 × D2 populations, respectively (Fig. 3, Appendix A5). Most *G. max* alleles at those QTLs had additive effects of decreasing PROD_1 (Add, –73 to –496 seeds; PVE, 8.8–33.8%), PROD_2 (Add, –5.1 to –42.5 g; PVE, 6.8–33.5%), PROD_4 (Add, –74 to –229 pods; PVE, 7.5–33%), PROD_5 (Add, –6.7 to –18.6 g; PVE, 10.2–28.8%), and PROD_6 (Add, –4 to –50 cm; PVE, 11.5–40.2%) and increasing PROD_3 (Add, +0.6 to 1.1 g; PVE, 7.8–28.2%).

The *G. max* alleles at several QTLs had effects opposite of those expected based on the parental phenotypes (Appendix A5), that is, in the W1 × D1 population, toward decreased PROD_3 for the QTL on LG-D1b and LG-M (Add, –0.2 to –0.3 g; PVE, 6.4–8.1%, south field), increased PROD_4 for the QTL on LG-M (Add, +158; PVE, 14.8%), and increased PROD_5 for the QTL on LG-M (Add, +6.6 g; PVE, 15.8%). In the W2 × D2 population, *G. max* alleles had effects toward decreased PROD_3 for the QTL on LG-C2 (Add, –1.3 g, PVE, 43.5%), increased PROD_5 for the QTL on LG-D2 and LG-M (Add, + 9.5 g; PVE, 22.6%), and increased PROD_6 at the QTL on LG-D2 (Add, +19.0 to +20.1 cm; PVE, 7.7–13.4%).

QTLs with large effect on seed production–related traits such as PROD_1, PROD_2, PROD_3, PROD_4, PROD_5, and PROD_6 were located near a marker *LFsoy3* in both the W1 × D1 and W2 × D2 populations (Fig. 3B). The *G. max* alleles at those QTLs, except for PROD_3, had additive effects of decreasing the phenotypic values for those traits, but the magnitude of effect differed depending on the test location (Appendix A5). For both populations, the additive effects for PROD_1, PROD_2, and PROD_5 were greater than those in the north field. Although the effects of QTLs for PROD_4 would be expected to be consistent, they did not seem to be related in the populations. The frequency of pods with only one or two seeds on plants in the north field was greater than for plants in the south field (data not shown), which may explain this inconsistency.

#### Flowering phenology

Four QTLs for flowering phenology were detected in each population (Fig. 3, Appendix A5). In general, *G. max* alleles at these QTLs had additive effects of hastening FLOW (Add, 0 to –3.5 days; PVE, 5.2–54.4%). In contrast, the *G. max* allele of the QTL on LG-D1b in the W1 × D1 population delayed FLOW (Fig. 3A). The location of the QTL identified on LG-L in the W1 × D1 population (Add, –1.8 to –2.4 days; PVE, 14.1–21.3%) was very near that of a QTL identified in the W2 × D2 population (Add, –0.8 day; PVE, 5.5% [Fig. 3B]). The locations of QTLs with large effects on FLOW were different between the two populations; in the W1 × D1 population, a major QTL was found on LG-O (Add, –2.8 to –3.5 days; PVE, 29.6–54.4%), whereas in the W1 × D1 population, a major QTL was found on LG-H (Add, –1.8 to –3.2 days; PVE, 26.6–36.8%).

### QTL analyses for BC_1_F_1_ populations

Linkage maps for W1 × D1 and W2 × D2 BC_1_F_1_ populations were constructed using 214 and 199 markers, respectively (Table 1, Fig. 3). In total, 8 and 20 QTLs were detected in the W1 × D1 and W2 × D2 populations, respectively (Fig. 3, Appendix A5).

#### Seed dormancy

Three QTLs for seed dormancy were detected in each population (Fig. 3A, Appendix A5). Although the QTLs for DORM_2 on LG-A2 were detected only in the W2 × D2 population, the QTLs for DORM_1 on LG-A2 were detected across combinations (W1 × D1 and W2 × D2), suggesting that the *G. max* alleles have a consistent genetic effect even within a high percentage of wild genetic background. The *G. max* allele at this QTL on LG-A2 had a large negative effect on DORM_1 (Add,–7% to –8%; PVE, 21.2–22.3%).

#### Seed production

Three and 15 QTLs related to seed production were detected in the W1 × D1 and W2 × D2 BC_1_F_1_ populations, respectively (Fig. 3, Appendix A5). No QTL was common between the F_2_ and BC_1_F_1_ generations. For traits PROD_1, PROD_2, PROD_4, and PROD_5, no QTLs were detected in the W1 × D1 population, but several QTLs different from those found in the F_2_ generation were identified in the W2 × D2 population. As in the F_2_ generation, most *G. max* alleles had the effect of decreasing seed production–related traits, that is, at QTLs for PROD_5 (Add, –8.6 to –11.6 g; PVE, 5.1–9.4%) and PROD_6 (Add, –21 to –62 cm; PVE, 7.5–20.2%). However, some *G. max* alleles at those QTLs from the W2 × D2 population had the effect of increasing PROD_1 on LG-A2 (Add, +354 seeds; PVE, 5.5%), PROD_2 on LG-A2 (Add, +17.9 g; PVE, 7.4%), PROD_4 on LG-E (Add, +113 pods; PVE, 5.1%) and on LG-M (Add, +112 pods; PVE, 4.9%), and PROD_6 on LG-A2 and LG-O (Add, +20 to +21 cm; PVE, 6.9–7.9%). The *G. max* alleles at all QTLs detected had the effect of increasing PROD_3.

#### Flowering phenology

Two QTLs for FLOW were detected in each population (Fig. 3B, Appendix A5). Although the QTLs in the two populations were different, the *G. max* allele at each one had the effect of delayed flowering time (FLOW, –1.8 to –5.0 days; PVE, 11.2–42.5%). There were QTLs in common between F_2_ and BC_1_F_1_ on LG-O in the W1 × D1 population (FLOW, –2.8 to –5.0 days; PVE, 29.6–54.4%) and on LG-H in the W2 × D2 population (FLOW, –1.8 to –3.2 days; PVE, 15.3–36.8%).

### QTL analyses for BC_2_F_1_ and BC_1_F_2_ populations

The linkage maps for W1 × D1 and W2 × D2 BC_2_F_1_ populations were constructed by using 103 and 72 markers, respectively (Table 1, Fig. 3). These markers were located in the heterozygous regions in the selected BC_1_F_1_ plants. In addition, BC_1_F_2_ populations were developed by using seeds from self-pollination of the two selected BC_1_F_1_ plants (W1 × D1 and W2 × D2) and partial linkage maps were constructed. The linkage maps for the W1 × D1 and W2 × D2 BC_1_F_2_ populations were constructed by using 105 and 72 markers, respectively. The order of markers in each linkage map was well conserved between the W1 × D1 and W2 × D2 populations as well as among the F_2_, BC_1_F_1_, and BC_2_F_1_ populations (Fig. 3A and B). Entire linkage groups (LG-A1, -C1, -I, and -M in W1 × D1 and LG-C1, -B2, -D2, -G, -J, and -O in W2 × D2) were found to have been replaced with *G. soja* genome in the two selected BC_1_F_1_ plants, BC_2_F_1_ population and BC_1_F_2_ population.

In the BC_2_F_1_ generation, which had a higher percentage of *G. soja* genetic background than the BC_1_F_1_, but included the selected fitness-related alleles from *G. max*, 10 QTLs were detected in both the W1 × D1 and W2 × D2 populations (Fig. 3, Appendix A5). Similar to the BC_1_F_1_ generation, most QTLs in the BC_2_F_1_ generation were different from those detected in the F_2_ generation. Unlike the situation in the BC_1_F_1_ generation, the effects of DORM_1 and DORM_2 QTLs on LG-A2 were not detected in W1 × D1 combination (Fig. 3A).

In the BC_1_F_2_ generation, which had a similar percentage of *G. soja* background to the BC_1_F_1_ generation but was homozygous for selected fitness-related alleles from *G. max*, 19 and 17 QTLs were detected in the W1 × D1 and W2 × D2 populations, respectively (Fig. 3, Appendix A5). The major QTLs for seed dormancy on LG-A2, C2, and D1b (Fig. 3A) and for seed number on LG-L (Fig. 3B) were well conserved between the F_2_ and BC_1_F_2_ generation, except for the DORM_1 QTL on LG-A2 in the W1 × D1 population, which was present in the F_2_ but not detected in the BC_1_F_2_ generation.

## Discussion

### Life history in relation to hybrid derivatives

In a previous study, hybrid derivatives that had arisen from gene flow between *G. soja* and *G. max* were grown in several natural habitats in Japan (Kuroda et al. 2010). Because the hardness of the seed coat, a phenotype related to seed dormancy (Table 4), is largely determined by the phenotype of the maternal *G. soja* plant, F_1_ seeds produced by pollen from *G. max* can survive in the soil several years, and the F_1_ plants can grow in the wild with *G. soja*. Here, FLOW of F_1_ hybrids tended to be similar to that of *G. soja* parent or intermediate between *G. soja* and *G. max* parent (Table 3), indicating that the flowering of natural F_1_ hybrids and local *G. soja* could overlap in several parts of Japan where natural hybrids have been identified. Due to genetic segregation in the F_2_ progenies, the extent of overlapping flowering time with *G. soja* will be reduced in that generation. However, once secondary gene flow from the F_1_ hybrid to *G. soja* has occurred, most of the backcross progenies are expect to have flowering time relatively similar to that of *G. soja* (Table 3). As the outcrossing rate in wild soybean populations has been reported to be 9.3–19% (Fujita et al. 1997) and 0–6.3% (Kuroda et al. 2008), our results suggest that *G. max* alleles can persist at some frequency in wild populations as long as gene flow continuously occurs at or near the maximum frequency.

Under the experimental field conditions, the total seed number (PROD_1) of the F_1_ hybrid was similar to or less than that of the corresponding *G. soja* parent (Table 3). PROD_1 of most F_2_ progenies was usually less than that of *G. soja*, although some F_2_ individuals revealed a similar or greater PROD_1 than the *G. soja* parent (Fig. 2A and B). As the proportion of *G. soja* background increased through backcrossing, the frequency of hybrid derivatives that revealed similar PROD_1 to *G. soja* also increased (Fig. 2C–F). However, after one round of self-pollination of the BC_1_F_1_ progenies, BC_1_F_2_ plants with short plant height and low seed production, as was seen in the F_2_ progenies, appeared again (Fig. 2G and H).

Most *G. max* seeds died in the soil during the winter, whereas the *G. soja* seeds survived (DORM_1, Table 3). Although DORM_1 of the F_1_ hybrids was intermediate between *G. max* and *G. soja*, F_2_ progenies revealed wide variation in DORM_1 (Fig. 2A and B). The extent of DORM_1 of the F_2_ progenies was related to the seed color (Table 4). As the proportion of *G. soja* background was increased by backcrossing with *G. soja*, the seed morphology (i.e., seed coat color and size) became closer to that of *G. soja*, and DORM_1 of the BC_1_F_1_ progenies increased (Fig. 2C–F). However, after one round of self-pollination of the BC_1_F_1_ progenies, BC_1_F_2_ seed/plants with low DORM_1 appeared (Fig. 2G and H). To understand this further, the phenotypic variation observed in the hybrid progenies was genetically dissected into QTLs by constructing genetic linkage maps.

### Seed dormancy–related QTLs

Seedling emergence represents the interface between two demographic events: seed production and seedling recruitment. Because seed dormancy–related traits determine the timing of seedling emergence, the physiology of seed dormancy has a large effect on fitness. Good water permeability is an important trait for uniform and rapid germination in *G. max* cultivation and food processing. Conversely, rapid water uptake is known to lead to cell damage in the cotyledon (Powell and Matthews 1978) and is disadvantageous to survival of *G. soja* during winter in natural habitats. The physiological difference has been characterized by many researchers who have measured traits such as seed water imbibition or seed hardness during several days under germinable conditions. However, evaluation of seed dormancy is generally quite different between artificial and natural conditions in terms of time, water, and temperature conditions. Even *G. max* seed, which imbibes water during winter, could survive winter in 2006 (Table 3), indicating that water imbibition does not always lead to loss of seed viability. In this study, three major QTLs affecting both DORM_1 and DORM_2, which are located on LG-A2, -C2, and -D1b (Fig. 3A), were generally consistent over generations and crossing combinations. A significant high correlation between DORM_1 and DORM_2 was observed (Appendix A4): seeds from hybrid derivatives that had *G. max* alleles at those QTLs imbibed water easily and appear to have rotted in the soil over the winter. In particular, the *G. max* allele for DORM_1 on LG-A2 was found to be partially dominant to the *G. soja* allele because its effect appeared in BC_1_F_1_ progenies and it had a large effect of reducing survival rate in the W2 × D2 population (Appendix A5B). Therefore, the effect of such strong *G. max* alleles may lead to reduced winter survival of the seeds produced by an F_1_ hybrid plant as well as by later-generation progenies.

Nevertheless, the magnitudes of allele effects at the three major DORM_1/DORM_2 QTLs were slightly different depending on the cross combination. For example, the effect of the QTL on LG-A2 was strongest among the three QTLs in the W2 × D2 population, whereas it was similar to that of the other two QTLs in the W1 × D1 population (Fig. 3A). This explains the different level of seed winter survival between the W1 × D1 and W2 × D2 combinations. All the previously reported QTLs had the effect of causing water imbibition when the alleles at those loci were from *G. max*. In a *G. max* × *G. soja* population, Keim et al. (1990) detected four QTLs on LG-A2, -L, and -D1b by evaluating imbibition of F_4_ seeds for 7 days at room temperature. In contrast, Sakamoto et al. (2004) and Liu et al. (2007) identified two QTLs, located in LG-C2 and -D1b, by evaluating imbibition of seeds for 12 h and 24 h at room temperature, respectively. *Glycine gracilis* is an intermediate form between *G. max* and *G. soja* that originated in northeastern China (Hymowitz 2004). Three QTLs (on LG-C2, -D1b, and -I) were identified in a *G. max* × *G. gracilis* population by testing imbibition of seeds for 24 h at 25°C (Watanabe et al. 2004). These results indicate that QTLs on LG-C2 and -D1b are common among *G. max* × *G. soja* populations, but that a QTL on LG-A2 is not consistently detected in such populations. Similarly, in this study, no QTL for seed hardness (DORM_2) was detected on LG-A2 in the W1 × D1 population (Fig. 3A). It is very interesting that QTLs for seed winter survival (DORM_1), which required a long-term evaluation in the field, were successfully identified in the W1 × D1 combination in approximately the same region on LG-A2 where QTLs for DORM_1 and DORM_2 were detected in the W2 × D2 combination. One possible explanation of this finding is that the effect of a QTL on LG-A2 may appear when seeds imbibe water during long-term evaluation if the seed coat of *G. max* has resistance to water imbibition. The slow imbibition rate seen for D1 parent also supports this explanation and suggests that there is allelic variation within *G. max* for a seed hardness QTL on LG-A2.

Based on the map locations of gene-derived markers and the magnitude of QTL effects, the DORM_1/DORM_2 QTLs on LG-A2 and LG-C2 are tightly linked to the *I* locus and *T* locus, respectively, and the genes responsible for DORM_1 are either *I* and *T* themselves or genes closely linked to those loci (Fig. 3A). The *I* allele, which suppresses seed coat pigmentation, is dominant to the *i* allele, and the *T* allele, which confers pigment pubescence, is dominant to the *t* allele (Bernard and Weiss 1973). Hybrid derivatives without black seed coat (i.e., those with the *I* allele) showed low seed survival (Table 4), and, thus, the *I* allele is related to the water imbibition ability of *G. max,* which might be due to a physical characteristic of the seed coat. Epistatic interaction between the *I* and *T* loci has been reported to cause seed coat cracking when the alleles at both *I* and *T* locus are recessive and homozygous (Lindstrom and Vodkin 1991). Such cracked F_3_ seeds produced from several F_2_ individuals imbibed water quickly and failed to survive during winter (Table 4). Thus, epistatic interactions account for the reduced fitness of progenies derived from self-pollination, in spite of a low proportion of double-recessive individuals in the progenies, through their influence on seed viability or survival.

### Seed production–related QTLs

The genes for domestication-related traits, which differentiate between crops and their wild relatives, are not randomly distributed across crop genomes (Ross-Ibarra 2005; Kaga et al. 2008). In this study, QTLs with high contributions to seed production–related traits, representing distinct differences between *G. soja* and *G. max*, tended to be concentrated in a particular genomic region on LG-L (Fig. 3B). Those QTLs were common between different cross combinations (W1 × D1 and W2 × D2) as well as across different generations. One possible reason for the positive, high correlation of total number of seed (PROD_1) with traits related to plant size such as stem dry weight (PROD_5) and stem length (PROD_6 [Appendix A4]) would be a gene related to stem elongation. Classically, stem termination in soybean is known to be controlled by two loci, *Dt*1 and *Dt*2 (Bernard 1972). The determinate stem type (*dt*1 allele) shows little growth in stem length after flowering, whereas the indeterminate stem type (*Dt*1 allele) continues to elongate even after flowering. An intermediate phenotype, called semideterminate, is conditioned by the *Dt*2 locus (Bernard 1972). Because *Dt*1 and *Dt*2 have been mapped on LG-L and LG-G, respectively (Cregan et al. 1999), the QTL with a strong contribution to stem length (PROD_6) on LG-L in this study is likely to be the *Dt*1 locus (Fig. 3B). Our results indicate that the *G. max* allele at this locus has the effect of reducing the number of seeds produced by hybrids between *G. max* and *G. soja*, as previously reported by Wang et al. (2004). Intriguingly, QTLs for seed weight (PROD_3) as well as other seed production–related traits were closely linked to marker *LFsoy*3, which was designed to detect a soybean homolog of *PsTFL1a*, a gene-controlling stem termination in *Pisum* (Foucher et al. 2003). Further studies are necessary to clarify the pleiotropic effect of soybean *TFL1a* on these traits. The *G. max* allele at the QTL for PROD_6 on LG-L was confirmed to have a moderate negative effect in the BC_1_F_1_ and BC_2_F_1_ populations, but it had no effect on PROD_1 as was found in the progenies from self-pollination (Fig. 3B, Appendix A5). A QTL for both PROD_6 and PROD_1 was identified again on LG-L in the BC_1_F_2_ population. These results indicate that the *G. max* allele is recessive to the *G. soja* allele because its effects were detected only in progenies generated by self-pollination.

### Flowering phenology–related QTL

Photosensitivity is also an important plant response that is heavily involved in the control of flowering as well as in successful seed production. There were clear differences between the W1 × D1 and W2 × D2 populations in terms of both days to first flower (FLOW) (Table 3). The W1 × D1 population, representing northern Japanese germplasm, had shorter FLOW than the W2 × D2 population, representing southern Japanese germplasm. This difference reflects the adaptive strategy of *G. soja* and *G. max* in Japan. In northern Japan, the growing season is relatively short; thus, the W1 × D1 population might respond to warm temperatures and start to produce seeds during the short period of moderate climate even if the plants are not large. In contrast, the W2 × D2 population might respond to photoperiod and start to produce seeds only after the plants have grown large because autumn is relatively long in southern Japan.

Based on the location of SSR markers linked to previously reported flowering loci, the FLOW QTLs on LG-O (W1 × D1 population), -L (W1 × D1 and W2 × D2 population), and -I (W1 × D1 population) found in this study (Fig. 3B) are thought to be the classical maturity loci *E*2 (Bernard 1971), *E*3 (Buzzell 1971), and *E*4 (Buzzell and Voldeng 1980). The other FLOW QTLs with a large effect (i.e., that on LG-H) or with a moderate effect (i.e., on LG-E and -F in the W2 × D2 population [Appendix A5B] and on LG-D1b and -K in the W1 × D1 population [Appendix A5A]) have not been previously described and might be new loci for flowering time in soybean. Although a QTL for days to flowering on LG-C2 has been reported in a *G. max* × *G. gracilis* population (Yamanaka et al. 2001; Watanabe et al. 2004) and in a *G. max* × *G. soja* population (Liu et al. 2007), no flowering time QTL at that location was consistently identified in this study.

### Evolutionary aspect of fitness-related QTLs and conclusions

Natural selection is expected to occur on the phenotypes of individuals that constitute *G. soja* populations, including hybrids between *G. soja* and *G. max*. Moreover, the phenotype of the hybrid progenies is influenced by the genetic variability of both *G. max* and *G. soja*, in response to a heterogeneous environment such as the natural habitat of *G. soja*. The results obtained here should be considered as an estimate obtained under conditions of maximum plant growth and seed production because the hybrid derivatives were widely spaced in the field (i.e., at intervals of 1 m); the results might have been different if the plants had been evaluated under conditions favoring high mortality of seedlings and restricted seed production in the competitive native weed population.

Genotype-dependent phenotypic response to different environments is common to quantitative traits and is referred to as phenotypic plasticity (Bradshaw 1965). In particular, the genes for wide adaptability that might have accumulated during human selection of *G. max* are probably different from those accumulated during ecological adaptation of *G. soja*, and they are likely to control more than the obvious morphological differences between the two species. For this reason, the effects of *G. max* genes were examined in this study in two types of hybrids between *G. soja* and *G. max* and were tested in two regions of Japan.

A large number of genes and their interactions with environmental changes during plant growth are thought to influence seed production. Nevertheless, the only QTL with a strong effect on PROD_1 between *G. soja* and *G. max* across different regions was the one identified on LG-L (Fig. 3B). The limited ability to detect QTLs involved in complex epistatic interactions might have led to underestimation of the number of loci involved in PROD_1 because QTLs for traits such as PROD_4 and FLOW that might be expected to affect PROD_1 were not always detected as QTLs for PROD_1.

Until recently, little has been known about the effect of *G. max* alleles within a predominantly *G. soja* genetic background. In this study, the genetic effects of those *G. max* alleles were not expressed as phenotypes in the BC_1_F_1_ and BC_2_F_1_ generations, indicating that most *G. soja* alleles are dominant to *G. max* alleles; one notable exception was the QTL for seed dormancy on LG-A2 (Fig. 3A). Snow et al. (1999) indicated that after two or three generation of backcrossing, hybrid derivatives in which crop alleles have been introgressed can be just as competitive and successful as wild plants. In this study, PROD_1 and DORM_1 in the BC_1_F_1_ and BC_2_F_1_ generation approached the values for *G. soja* as the proportion of *G. soja* genetic background increased (Table 3). Although QTLs at which *G. max* alleles had the increasing effect on PROD_1 and DORM_1 were not consistent over generations and crossing combinations (Fig. 3, Appendix A5), these alleles may have the potential to increase the fitness of hybrid derivatives. Individual plants that had higher fitness than *G. soja* in terms of SURV could be found in most generations of both the W1 × D1 and W2 × D2 populations (Fig. 2).

In contrast, QTLs at which *G. max* alleles had negative effects on fitness were consistently detected in both cross combinations and in different generations. In particular, QTLs for DORM_1 on LG-A2, -C2, and -D1b (Fig. 3A) and for PROD_1 on LG-L (Fig. 3B) were found in both cross combinations. This is one reason why hybrid derivatives do not survive in natural habitats (Kaga et al. 2005; Kuroda et al. 2005, 2006b, 2007), and why genetic differentiation is maintained between *G. soja* and *G. max* (Maughan et al. 1996; Powell et al. 1996; Xu and Gai 2003; Kuroda et al. 2006a). Previously, it was reported that hybrids between wild and crop species should be less fit than their wild parents due to the burden that crop traits would introduce into wild plants (De Wet and Harlan 1975). Current knowledge of the genetic basis of domestication traits suggests that few genomic regions are usually involved in domestication (White and Doebley 1998; Gross and Olsen 2010); thus, these regions could be purged quite rapidly with no long-term impact on fitness within the first few generations after hybridization. Our results support these studies and suggest that the risk of transgene dispersal into the wild soybean gene pool is generally low in Japan. The simulation studies as to what extent *G. max* alleles persist under a mixed mating system (i.e., considering the relative proportions of progenies both from self-fertilization and from outcrossing events) is required to improve the assessment of environmental transgene dispersal from GM soybeans.

## Supporting Information

**Appendix A1.** Geographical locations of experimental fields.

**Appendix A2.** Meteorological data for the north (Akita), central (Ibaraki), and south (Hiroshima) fields for 2005, 2006, and 2007, and mean values for the years 1979–2000.

**Appendix A3.** Primer sequences for five markers.

**Appendix A4.** Correlations among fitness-related traits for both W1D1 and W2D2 hybrids.

**Appendix A5A.** QTLs detected in F_2_, BC_1_F_1_, BC_1_F_2_, and BC_2_F_1_ generations of the W1 × D1 population.

**Appendix A5B.** QTLs detected in F_2_, BC_1_F_1_, BC_1_F_2_, and BC_2_F_1_ generations of the W2 × D2 population.
