# Supplementary material for: QTL affecting fitness of hybrids between wild and cultivated soybeans in experimental fields
Source: Ecol Evol. 2013 Jun 5;3(7):2150–68. doi: 10.1002/ece3.606 (PMC3728954; doi:10.1002/ece3.606)
Supplement: Supplementary file 1 [file ece30003-2150-SD1.docx]

**Supporting Information**

Appendix 1. Geographical locations of experimental fields.

Appendix 2. Meteorological data for the north (Akita), central (Ibaraki), and south (Hiroshima) fields for 2005, 2006, and 2007, and mean values for the years 1979–2000.

Appendix 3. Primer sequences for five markers.

Appendix 4. Correlations among fitness related traits for both W1D1 and W2D2 hybrids.

Appendix 5a. QTLs detected in F_2_, BC_1_F_1_, BC_1_F_2_, and BC_2_F_1_ generations of the W1xD1 population.

Appendix 5b. QTLs detected in F_2_, BC_1_F_1_, BC_1_F_2_, and BC_2_F_1_ generations of the W2xD2 population.
